# Nitrite supplementation alleviates cerebrovascular dysfunction in chronically stressed mice, but cognitive decline remains

**DOI:** 10.1113/EP092501

**Published:** 2025-07-23

**Authors:** Emily Burrage, Tyler Coblentz, Saina S. Prabhu, Nicole M. Eminhizer, Ryan Childers, Randall W. Bryner, Sara E. Lewis, Brooke A. Maxwell, Evan R. DeVallance, Eric E. Kelley, Paul D. Chantler

**Affiliations:** ^1^ Department of Neuroscience West Virginia University School of Medicine Morgantown West Virginia USA; ^2^ Division of Exercise Physiology West Virginia University School of Medicine Morgantown West Virginia USA; ^3^ Department of Pharmaceutical Sciences West Virginia University School of Medicine Morgantown West Virginia USA; ^4^ Department of Physiology and Pharmacology West Virginia University School of Medicine Morgantown West Virginia USA

**Keywords:** cognition, middle cerebral artery, oxidative stress, sodium nitrite, UCMS

## Abstract

This study aimed to determine whether sodium nitrite supplementation prevented chronic stress‐induced cerebrovascular dysfunction and cognitive decline. We hypothesize that nitrite supplementation will prevent the oxidative environment and cerebrovascular dysfunction associated with chronic stress and maintain cognitive health. Eighteen‐week‐old male/female C57BL/6 mice underwent 8 weeks of control conditions or unpredictable chronic mild stress (UCMS) with or without sodium nitrite (50 mg/L) in the drinking water. Excised middle cerebral arteries (MCA) were mounted in a pressurized myobath and exposed to increasing concentrations of acetylcholine (ACh). Nitrite supplementation prevented the UCMS‐induced impaired ACh response in the MCA. We examined xanthine oxidoreductase (XOR) as a potential mechanism by determining XOR protein abundance, activity, and hydrogen peroxide production in the liver and brain. Nitrite supplementation prevented the development of an oxidative environment within the liver, brain and cerebrovasculature. Assessment of working memory revealed that sodium nitrite did not fully prevent the impairment of cognitive function because of chronic stress. These data suggest that nitrite supplementation protects against stressed‐induced cerebrovascular dysfunction by limiting the actions of oxidants, potentially via XOR, while improving NO bioavailability. However, nitrite was not sufficient to prevent cognitive impairment with chronic stress.

## Abstract (highlights)

- **What is the central question of this study?**
  Can chronic supplementation of NO_2_^−^ during chronic stress preserve middle cerebral arteries endothelial function and cognitive health by maintaining normal levels of NO bioavailability and oxidative stress?
- **What is the main finding and its importance?**
  Nitrite supplementation protects against stressed‐induced cerebrovascular dysfunction via oxidants, potentially via xanthine oxidoreductase, while improving NO bioavailability. However, nitrite was not sufficient to prevent cognitive impairment with chronic stress.

## 1 INTRODUCTION

Chronic stress is a serious health and social issue (Tong et al., 2019), affecting more than 76% of adults (Bethune, 2022). Chronic stress in early life (Miller et al., 2011) and adulthood (Steptoe & Kivimaki, 2013; Toren et al., 2014) is linked to increased cardiovascular (CV) risk, even when adjusted for other CV risk factors (Kivimaki et al., 2012). However, the cellular underpinning of this relationship is poorly understood.

Our work, along with others, shows a clear link between psychosocial stress and impaired vascular function in clinical and pre‐clinical models (Branyan et al., 2018; Brooks, Hileman, Chantler, et al., 2018; DeVallance et al., 2021; Greaney et al., 2019). However, these effects are most profound in the cerebrovasculature (Brooks, Branyan, DeVallance, et al., 2018; Brooks, Hileman, Chantler, et al., 2018; Burrage, Coblentz, Prabhu, et al., 2023). This is important because cerebrovascular dysfunction can lead to reduced nutrient supply (O_2_, and glucose) (Harper et al., 1984) to neural tissue and may exacerbate psychological stress or predispose individuals to cognitive decline and dementia (Dichgans & Leys, 2017; Burrage, Coblentz, Prabhu, et al., 2023). This is supported by an epidemiological link between stress and cognitive performance from the REGARDS study (Kulshreshtha et al., 2023).

Mechanistically, oxidant stress diminishes NO bioavailability, leading to cerebrovascular dysfunction (Brooks, Branyan, DeVallance, et al., 2018; Burrage, Coblentz, Prabhu, et al., 2023; Chantler et al., 2015; De Silva et al., 2018). Xanthine oxidoreductase (XOR) is a prominent source of vascular oxidants as it is expressed in endothelial cells and actively released into circulation by hepatocytes (Cantu‐Medellin & Kelley, 2013; DeVallance et al., 2023; Kelley et al., 2010; Schmidt et al., 2023). Conversely, in the presence of abundant nitrite/nitrate XOR function can be shifted towards its nitrate reductase activity (Cantu‐Medellin & Kelley, 2013; Kelley, 2015; Li et al., 2005, 2009). This is potentially beneficial on two fronts: (1) generation of NO, and (2) it prevents electron donation to molecular oxygen and oxidant formation. Therefore, this study aimed to build upon our previous work (Burrage, Coblentz, Prabhu, et al., 2023) and determine if nitrite (NO_2_^−^) supplementation concurrently with exposure to chronic stress using the unpredictable chronic mild stress (UCMS) model could protect cerebrovascular function and cognitive health. Further, as XOR catalyses the reduction of NO_2_^−^ to NO, we explored XOR as a potential mechanism of action of NO_2_^−^ supplementation. We hypothesized that chronic supplementation of NO_2_^−^ during the chronic stress model will maintain normal levels of NO bioavailability and oxidative stress while preserving middle cerebral artery (MCA) endothelial function in chronically stressed mice.

## 2 METHODS

### 2.1 Animals

#### 2.1.1 Ethical approval

All experiments were reported in accordance with the ARRIVE (Animal Research: Reporting in vivo Experiments) guidelines (Percie du Sert et al., 2020). The protocol (ACUC#1603000971) received prior approval from the WVU HSC Animal Care and Use Committee as per the National Institutes of Health Office of Laboratory Animal Welfare guidelines. Mice were housed at the West Virginia University Health Science Center animal husbandry, and had access to housing, food, and water ad libitum. Temperature in the husbandry was 20–24°C, and humidity was in the range 45–55%. The mice were exposed 12–12 h light–dark cycle, with lights turning on at 06.00 h.

#### 2.1.2 Animals and group allocation

Male and female C57BL/6J mice (stock no. 000664) were obtained from The Jackson Laboratory (Bar Harbor, ME, USA) at 15–16 weeks of age, and they were allowed to acclimate to the new environment for at least a week. At 18 weeks of age, all animals were singly housed and randomly assigned to one of the following groups for 8 weeks: (1) control (non‐UCMS), (2) UCMS, (3) control and nitrite (Con + NO_2_^−^), and (4) UCMS and nitrite (UCMS + NO_2_^−^). Once on protocol, all mice were housed overnight in the same OLAR room and handled daily. The UCMS groups were moved into the UCMS room, where they underwent the chronic stress paradigm. The control mice were also moved to a different room, similar in size, and remained under control conditions, allowing all mice to have similar exposure to experimenter intervention. At 6 months of age, mice were euthanized under 4–5% isoflurane to induce anaesthesia, then anaesthesia was maintained with 1.5% isoflurane while a thoracotomy was performed, and a blood sample was collected via cardiac puncture. Then the diaphragm was cut and the heart removed. At this point, the head was separated from the body and the brain removed from the skull (and processed as described below). A total of 146 male/female C57BL/6J mice (∼36 mice/group) aged 15–16 weeks were used in this study. To ensure we had sufficient tissues to perform the microvessel isolation, the separation of brain tissue (western blots, HPLC and coumarin boronic acid assay), and the behavioural analysis, we added extra cohorts over the year, whereby four mice per new cohort were randomly allocated to one of the four groups. No mice were lost due to the UCMS protocol, and any variability in numbers is attributed to experimental or molecular issues.

#### 2.1.3 Nitrite treatment

Vehicle (standard drinking water) or nitrite (NNaO_2_, Thermo Fisher Scientific, Waltham, MA, USA) was delivered daily in the drinking water at a concentration of 50 mg/L. The water was measured frequently to calculate the concentration of NO_2_^−^ consumed daily (∼5–6 mg/kg per day).

#### 2.1.4 Chronic stress intervention

We used the UCMS model to induce chronic stress in our mice. Every single stressor (damp bed, cage tilt, cage reversal, etc.) is of minor concern, like humans with one bad day among many average days. But with multiple stressors over 8 weeks, rodents cannot adapt, increasing stress hormones and limited grooming. This is like a human facing a wide variety of unpredictable hassles across many bad days. All mice were singly housed. In UCMS groups, all mice were moved out of their home cages and were exposed to the following mild environmental stressors in randomly chosen sequences for ∼7 h each day, 5 days/week, over 8 weeks with ∼3–4 stressors/day lasting for 1–3 h/stress. At the end of the stress days the mice were returned to their home cages.

1. *Damp bedding* – approximately 300 mL of water was added to each standard cage.
2. *Bath* – all bedding was removed and ∼1.3 cm of water was added to the empty cage. The water temperature was room temperature, ∼24°C.
3. *Cage tilt* – cage was tilted to 45 degrees without bedding.
4. *Social stress* – each mouse was switched into a cage with a neighbouring mouse.
5. *No bedding* – all bedding was removed from the cage.
6. *Alteration of light/dark cycles* – turning lights off/on in random increments for a scheduled period.

#### 2.1.5 Stress markers

Plasma collected during the terminal procedures was used to measure corticosterone using a commercially available ELISA kit (Arbor Assays, Ann Arbor, MI, USA, cat. no. K014). Corticosterone levels were examined in triplicate according to the manufacturer's instructions. During the 8‐week protocol, the rodents’ coats were evaluated. Each week the mice were inspected for grooming habits (Yalcin et al., 2005) and the total cumulative coat score was computed by giving an individual score of 0 (clean) or 1 (dirty) to eight different body parts (i.e., head, neck, back, forelimbs, stomach, hindlimbs, tail, genitals). Following the 8‐week protocol, sucrose splash testing was performed. The sucrose splash test consisted of spraying a 10% sucrose solution on the dorsal coat of a mouse in an empty cage; latency to begin grooming, as well as frequency, was recorded for a total of 5 min (Frisbee et al., 2015).

### 2.2 Behaviour

Behavioural assessments were conducted 1 day after the completion of the UCMS intervention. On Day 1, the Open Field Test was performed between 07.00 and 11.00 h under normal lighting conditions. One mouse per group (control, control + nitrite, UCMS, and UCMS + nitrite) was tested simultaneously, with randomized box assignments and thorough cleaning between animals. On Day 2, the Y‐maze test was conducted during the same light phase (07.00 to 11.00 h) but under dim lighting conditions. As only one apparatus was available, animals were tested randomly, ensuring at least one mouse from each group was tested per hour to minimize cortisol‐related circadian confounds. Euthanasia was performed the week following all behaviour testing.

#### 2.2.1 Open field test

This task evaluates locomotor activity and emotional reactivity (Denenberg, 1969). Total movement in the arena indicates general locomotor ability, and movement in the centre versus periphery indicates anxiety status. The apparatus consisted of a clear plastic box (40 cm × 40 cm) surrounded by a 16 × 16 laser beam array (San Diego Instruments, San Diego, CA, USA) that allowed for the assessment of movement in the horizontal axis. A second array placed 3.8 cm above the first beam allowed for the assessment of locomotion in the vertical axis (rearing behaviour). Following a 30‐min acclimation to the testing room (approximately 1500 lx), each subject was placed in the centre point of the arena and allowed to freely explore for 30 min. To assess treatment‐induced alterations in anxiety‐like behaviour, the open field arena was divided into a central square zone (approximately 700 cm^2^) and a peripheral frame (approximately 940 cm^2^) zone, and horizontal locomotion was assessed in each zone (peripheral and central). Thus, the dependent variables were the number of total horizontal movements via beam breaks in the arena as well as in the peripheral and central zones, and the number of vertical beam breaks (rearing). The 30‐min phase was also broken down into six 5‐min intervals, which, when analysed individually, did not reveal any differences.

#### 2.2.2 Y‐maze spontaneous alternation

To assess short‐term working memory, following a 30‐min room acclimation period, under indirect dim illumination conditions (approximately 150 lx), mice were placed in the centre of the three‐armed, Y‐shaped apparatus (approximate arm length = 38 cm, width = 8.25 cm, height = 13.25 cm) and allowed to freely explore for 8 min. Movement in the apparatus was recorded using the Anymaze tracking software (Stoelting, Chicago, IL, USA). The innate tendency of mice to spontaneously switch between the three arms and enter the least recently visited arm was assessed by determining the percentage of successful alternations ((number of correct alternations)/(total arm entries − 2) × 100). Other dependent variables recorded included distance moved (m) and speed (m/s). Between each animal, each apparatus was cleaned of debris and olfactory cues using an anti‐bacterial disinfectant (Virkon, Pharmacol, Waterbury, CT, USA).

### 2.3 Vascular reactivity/mechanics

Mice were euthanized, and the brain was carefully removed from the skull and placed in a cold physiological salt solution (PSS; 4°C). Both middle cerebral arteries (MCA) were dissected from their origin at the circle of Willis and placed into an isolated microvessel chamber (living systems) filled with PSS. Each MCA was subsequently doubly cannulated within a heated chamber (37°C) that allowed the lumen and exterior of the vessel to be perfused and superfused, respectively, with PSS from separate reservoirs. The PSS was equilibrated with a 21% O_2_, 5% CO_2_, and 74% N_2_ gas mixture and had the following composition (mM): 119 NaCl, 4.7 KCl, 1.17 MgSO_4_, 1.6 CaCl_2_, 1.18 NaH_2_PO_4_, 24 NaHCO_3_, 0.026 EDTA, and 5.5 glucose. Any side branches were ligated. MCA diameter was measured using television microscopy and an on‐screen video micrometer.

Measurements of vascular reactivity in isolated MCA were as follows. Following cannulation, MCAs were extended to their in situ length and were equilibrated at 70 mmHg mean arterial pressure. Active tone at the equilibration pressure was calculated as (Δ*D*/*D*_max_) × 100, where Δ*D* is the diameter increase from rest in response to calcium (Ca^2+^)‐free PSS, and *D*_max_ is the maximum diameter measured at the equilibration pressure in Ca^2+^‐free PSS. Following equilibration, the MCA dilator reactivity was assessed in response to increasing concentrations of acetylcholine (ACh, 10^−9^ M–10^−4^ M) to reflect endothelial‐dependent dilatation (EDD) and increasing concentrations of sodium nitroprusside (SNP 10^−9^ M–10^−4^ M) to reflect endothelial independent dilatation (EID), and a potent vasoconstrictor (phenylephrine, PE, 10^−9^ M–10^−4^ M). To assess the effects of NO on EDD, the MCA was acutely incubated (30 min) with l‐*N* ^G^‐nitro‐arginine methyl ester (L‐NAME, 10^−4^ M), and the MCA EDD response to ACh was repeated. To assess the acute effects of oxidative stress on modulating EDD, we acutely (30 min) incubate the MCA with (1) 4‐hydroxy‐TEMPO (TEMPOL, 10^−4^ M, Sigma‐Aldrich, St Louis, MO, USA, cat. no. 176141); and (2) febuxostat (10 mM; Axon Medchem BV, Groningen, Netherlands). Febuxostat is a specific XOR inhibitor that effectively blocks substrate access to the active site (Okamoto & Nishino, 2008; Okamoto et al., 2003), and completely inhibits endothelial cell‐bound XO‐derived oxidant formation (Malik et al., 2011), and chronic delivery of 10 mM of febuxostat in the drinking water drastically reduces XOR activity and H_2_O_2_ in various tissues (Burrage, Prabhu, Childers, et al., 2023).

After completing all procedures, the perfusate and superfusate were replaced with Ca^2+^‐free PSS, and the passive diameter of the fully relaxed MCA was determined over an intralumenal pressure range of 5–140 mmHg at 20‐mmHg pressure increments. After 7 min at each intraluminal pressure, the inner and outer diameters of the passive MCA were determined. Media thickness, lumen and outer diameters, and vessel cross‐sectional areas (used as indicators of structural alterations to the individual microvessel) were determined as follows: media wall thickness (WT, µm) = outer diameter − lumen diameter (i.e., OD − LD); media‐to‐lumen (M:L) ratio = MT/LD; cross‐sectional area (µm^2^) = outer vessel area − lumen area. Myogenic tone was assessed using the formula 1 − (active diameter/passive diameter) × 100. NO‐dependent dilatation was determined from the maximal EDD in the absence or presence of L‐NAME according to the following formula: NO‐dependent dilatation (µm) = maximum dilatation_ACh_ − maximum dilatation_ACh+L‐NAME._

#### 2.3.1 Cerebral microvessel isolation

To explore the vascular signalling pathway, the mouse brain was carefully removed, the MCA was dissected for pressure myography, and then the remaining cerebral microvessels (parenchymal and meningeal vessels) were isolated from the brain using the dextran centrifugation method. In brief, brain tissue was homogenized in 1× PBS and centrifuged 1078 g at 4°C. The supernatant, containing parenchymal tissue was discarded; the pellet was resuspended and layered over 15% dextran (in 1× PBS) (Sigma‐Aldrich, cat. no. 31390). Following 30‐min centrifugation at 1968 g at 4°C, the supernatant was discarded, and the pellet was resuspended in 1% bovine serum albumin (BSA) in 1× PBS. The suspension was then passed through a 70 µm cell strainer with the microvessels collected on the mesh. The microvessels were then washed with 1% BSA in 1× PBS and collected by centrifugation at 1107 g for 10 min at 4°C. Following this, microvessels were rinsed with 1× PBS, centrifuged at 1968 g  for 5 min at 4°C, and flash frozen for future use in other assays (adapted from Austin & Katusic (2020).

### 2.4 Tissue oxidants

As briefly described below, the brain, liver and microvessels were explored for oxidative products.

#### 2.4.1 XOR

The term XOR is a collective name for two isoforms of the same enzyme, xanthine dehydrogenase (XDH) and xanthine oxidase (XO). XOR is transcribed and translated as XDH, and conversion to XO depends upon post‐translational modification via oxidation of two critical cysteines or limited proteolysis. Both XDH and XO catalyse purine oxidation at their respective molybdenum cofactors; however, XDH utilizes NAD^+^ (NAD^+^ → NADH) as an electron acceptor, whereas XO utilizes O_2_ (O_2_ → O_2_^•−^ + H_2_O_2_). The bulk of intracellular XOR is found in the XDH configuration, whereas extracellular XOR is mostly XO (Cantu‐Medellin & Kelley, 2013; Kelley et al., 2010).

#### 2.4.2 Xanthine oxidase – HPLC

XO activity was measured in the plasma, liver and brain tissue as previously described using high‐performance liquid chromatography (HPLC) (Harmon et al., 2019; Schmidt et al., 2021). Samples were homogenized in ice‐cold RIPA buffer containing a protease inhibitor cocktail (Sigma‐Aldrich) and spun down. Tissue samples were processed as described below with a sample volume of 10 µL. Uricase was inhibited by oxonic acid (100 µM) to avoid an underestimation of enzyme activity. Total XOR activity was determined based on the rate of uric acid production in the presence of xanthine (75 µM) and nicotinamide adenine dinucleotide (NAD^+^, 0.5 mM). Allopurinol (100 µM), an inhibitor of XOR, was used in parallel samples to confirm that urate formation was specific. After 60 min of incubation at 37°C, the reaction was terminated by protein precipitation with cold acetonitrile. The samples were then centrifuged for 12 min at 13,200 *g*, at 4°C. Following centrifugation, the supernatant was removed and placed in borosilicate glass tubes. After a 60‐min dry‐down process, the samples were resuspended in isocratic mobile phase (300 µL) and filtered through a 0.20 µm nylon membrane filter unit into 11 mm plastic snap‐top auto‐sampler vials. The uric acid content of protein‐free samples was determined by an HPLC‐based electrochemical technique as described below. One unit of activity (U) is defined as 1 µmol/min urate formed at 37°C and pH 7.4. Total protein concentration was determined by the bicinchoninic acid (BCA) method (Thermo Fisher Scientific, Waltham, MA, USA). Measurements were carried out blindly without knowledge by the operator of the animal treatment groups. Uric acid was measured by electrochemical detection (UltiMate 3000 ECD‐3000RS, Thermo Fisher Scientifc, Waltham, MA, USA) coupled to reverse‐phase HPLC using a Phenomenex column (Luna 3 µm C18(2) 100 A, 150 × 4.6 mm, Torrance, CA, USA) and isocratic mobile phase (50 mM sodium dihydrogen phosphate, 4 mM dodecyltrimethylammonium chloride, 2.5% methanol, pH 7.0). Potentials for three channels were as follows: −100, 30 and 390 mV. Quantitation/integration was performed on the dominant peak. In the case of XO activity in plasma, 10–20 µL of plasma was used instead of tissue and processed with the same protocol as above, without gel filtration. XO activity in plasma utilized volume (U/mL) was the denominator rather than protein.

#### 2.4.3 Xanthine oxidoreductase – western blots

Liver and brain tissue were homogenized in 1× PBS plus HALT inhibitor and then centrifuged at 1107 g at 4°C for 20 min. An equal amount of protein for each sample was loaded into a polyacrylamide gel. After the completion of gel electrophoresis, the protein was transferred to nitrocellulose membranes overnight. XOR expression was assessed using an XOR antibody (1:500; Santa Cruz Biotechnology, Dallas, TX, USA; cat. no. sc‐398548) followed by a secondary antibody (1:10,000; LI‐COR, Lincoln, NE, USA; cat. no. 925‐32201 and 925‐68071). Glyceraldehyde 3‐phosphate dehydrogenase (GAPDH) (1:1000; Cell Signaling Technology, Danvers, MA, USA; cat. no. 2118) was used as a loading control. Blots were imaged using LI‐COR Odyssey and band intensity was assessed using ImageJ software.

#### 2.4.4 Coumarin boronic acid assay

Hydrogen peroxide (H_2_O_2_) abundance was measured in homogenate from the liver, brain and brain microvessel using the coumarin boronic acid (CBA) assay (Cayman Chemical Co., Ann Arbor, MI, USA cat. no. 14051) as previously described (DeVallance et al., 2019). In brief, 10 µg of lysates was loaded, in triplicate, into wells of a 384‐well black‐sided clear bottom plate. Subsequently, assay buffer composed of Hanks' balanced salt solution (HBSS) supplemented with 25 mM HEPES, 1% BSA, 10 µM DTPA (Diethylenetruaminepentaacetic acid), 100 µM L‐NAME, 1 mM taurine was added, and then CBA was added to each well at a final concentration of 0.5 mM. One well from each biological sample received an additional 1 kU/mL bovine liver catalase to act as a negative control. Upon addition of the CBA, plates were placed in a BioTek plate reader preheated to 37°C and read kinetically at excitation 350 nm and emission 450 nm. The average rate of fluorescence was determined over the linear portion of the response and then normalized by subtracting the rate of fluorescence from the negative control.

#### 2.4.5 Assessment of NO production

NO was determined by the accumulation of NO_2_^−^, the stable oxidation product of NO, in aqueous medium. NO was detected by chemiluminescence following the reduction of NO_2_^−^ with KI and CuSO_4_, according to the manufacturer's instructions (Sievers, GE Analytical Instruments, Cincinnati, OH, USA) (Khoo et al., 2012, 2015). Plasma was washed twice with HBSS at 37°C before incubation with 0.75 mL of HBSS containing 25 µM l‐arginine plus the addition of NO_2_‐ Fatty Acids (FA), native FAs, or vehicle (methanol) for 5–120 min. Following treatment, the HBSS plus treatment was collected from each well and centrifuged at 1000 *g* for 10 min. The resulting supernatant was then injected (50 µL via a Hamilton syringe) into the Sievers nitric oxide analyser (NOA). Each treatment was performed in triplicate and normalized to protein content. A serial dilution of nitrite standards was prepared for each experiment using a freshly prepared NaNO_2_ solution. Background levels of NO_2_^−^ concentrations were determined using samples of dH_2_O and HBSS containing l‐arginine.

### 2.5 Statistics

Data are presented as means ± SD, unless otherwise stated. Normality was evaluated by the Kolmogorov–Smirnov test. Power analysis was performed using PASS 15 software, and based on preliminary data the sample size was calculated based on the vascular outcomes and powered off max MCA EDD (2 × 4 × 2 factorial ANOVA, 99% power, α = 0.05), a sample size of 8/sex/group (2 conditions: non‐UMCS vs. UCMS; 4 groups: Con, Con + NO_2_^−^, UCMS, UCMS + NO_2_^−^; sex: male vs. female) would provide 99% power to find a significant difference in EDD among groups. To ensure we had sufficient tissues to perform the microvessel isolation, the separation of brain tissue (for western blots, HPLC and CBA assay) and the behavioural analysis, we added extra cohorts over the year and randomized them into one of four groups. Total mouse numbers/groups are detailed in the figure legends. As no statistical differences were noted between male and female mice for all variables, male and female data were combined for statistical approaches. During data collection, groups were unknown to the experimenter. The MCA maximal reactivity, MCA remodelling and molecular approaches were analysed by a two‐way (UCMS and NO_2_^−^) analysis of variance (ANOVA) with an interaction term, and Tukey's *post hoc* test was performed to determine differences among groups. The clinical characteristics, behavioural tests and the effects of acute TEMPOL, febuxostat and L‐NAME on the maximal dilatation of the MCA were examined with a two‐way ANOVA or a repeated‐measures ANOVA with an interaction term, and Tukey's *post hoc* test was performed when appropriate. Data analysis and graphing were conducted using GraphPad Prism version 9 software (GraphPad Software, Boston, MA, USA), and *P* ≤ 0.05 was set for statistical significance.

## 3 RESULTS

### 3.1 Animal characteristics and stress response

After the study, the only statistically significant difference in body mass was between UCMS and Control + NO_2_^−^ groups (Table 1). Plasma levels of corticosterone were significantly increased in the UCMS (95–105%) compared to the control and Control + NO_2_^−^ groups (*P *< 0.05). The plasma corticosterone levels did not reach statistical significance between UCMS and UCMS + NO_2_^−^ groups (*P* = 0.0598). Behavioural assessments revealed poorer coat status of mice undergoing UCMS as well as an increased latency to lick and fewer licks in the sucrose splash test compared to control mice (*P *< 0.05). Except for corticosterone, all other stress behaviours remained elevated in the UCMS + NO_2_^−^ group compared to the control group. There was a significant group by drug interaction for the plasma levels of nitrate/nitrite, and nitrate/nitrite was increased in the UCMS + NO_2_^−^ compared to the UCMS group (*P* = 0.03), whereas plasma levels of nitrate/nitrite were similar between other groups.

**TABLE 1:** Animal characteristics.

	Control	Control + NO_2_^−^	UCMS	UCMS + NO_2_^−^
Male (%)	60%	55%	52.5%	51%
Body mass (g)	27.1 ± 4	27.9 ± 3.4	25.5 ± 3.3^†^	26 ± 3.4
Mean blood pressure (mmHg)	119 ± 7.7	115 ± 8.9	124 ± 9.8	120 ± 6.9
Water intake (mL/day)	5.3 ± 2	4.7 ± 0.3	4.4 ± 1.3	5.6 ± 0.5
Plasma nitrate/nitrite	17.85 ± 11.07	15.82 ± 5.51	14.29 ± 7.49	32.24 ± 17.83^‡^
Corticosterone, (ng/mL)	112.3 ± 78.9	118 ± 37.2	230.4 ± 132*^†^	133 ± 59.4
Coat scores, AU	0.7 ± 1.2	1.3 ± 1.3	6.1 ± 1.4*^†^	6.2 ± 1.4*^†^
Sucrose splash test				
Latency	8.5 ± 7	12.5 ± 10	19.5 ± 17*	17.3 ± 16*
No. of licks	43.7 ± 19	36.7 ± 19	24.7 ± 15*^†^	24.4 ± 15*^†^

*Note*: Data are presented as means ± SD. *P < 0.05 vs. Control, ^†^ *P *< 0.05 vs. Con + NO_2_^−^, ^‡^ *P *< 0.05 vs. UCMS. *n* = 16–20/group except for corticosterone, blood pressure and nitrate/nitrite, *n* = 8–12/group. Plasma NO_2_^−^ showed a significant group by drug interaction. Abbreviation: UCMS, unpredictable chronic mild stress.

### 3.2 MCA function and wall mechanics

**FIGURE 1:**
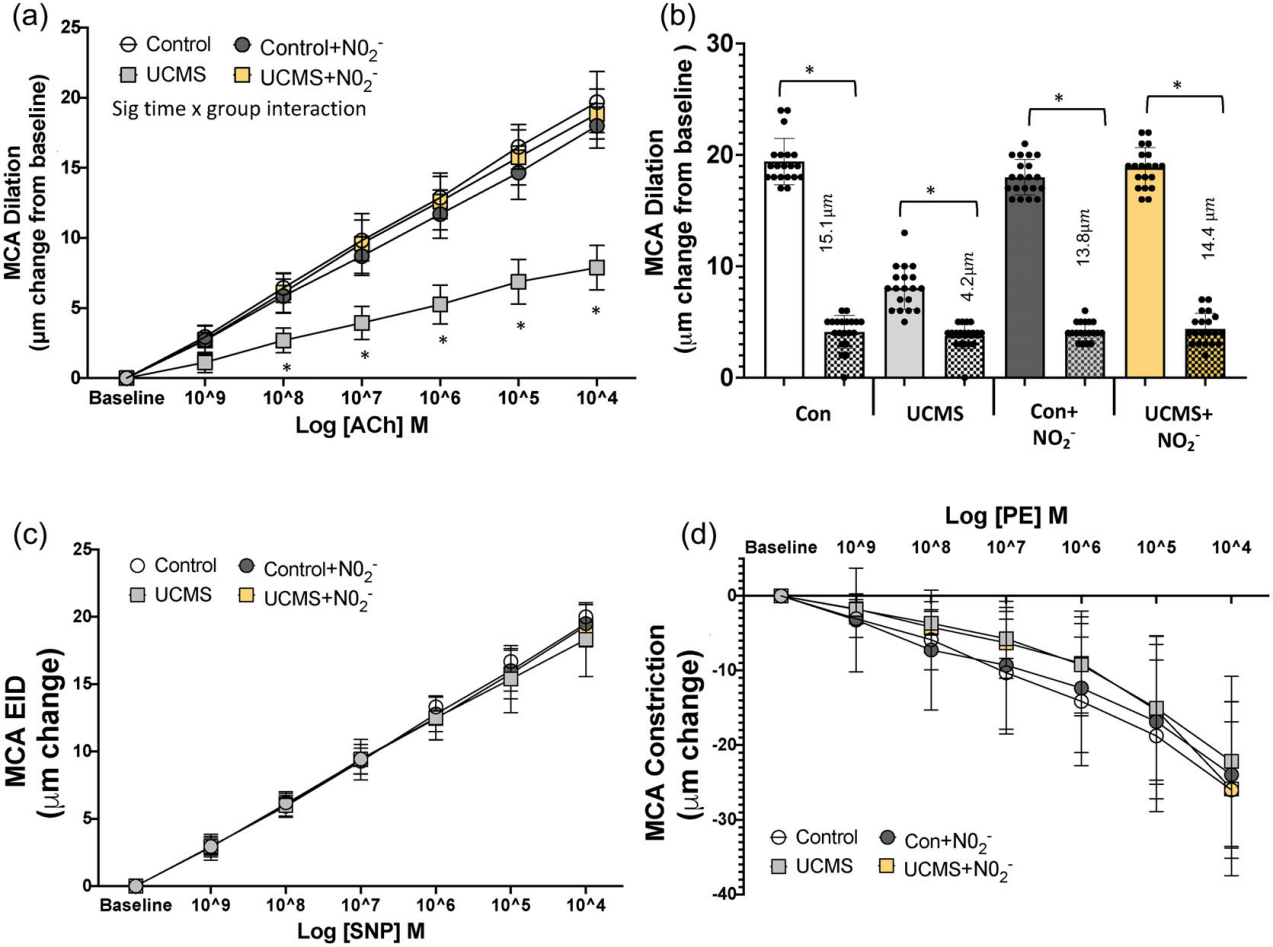
Cerebrovascular function. (a) Middle cerebral artery (MCA) dose–response curve for endothelial‐dependent dilatation (EDD) to acetylcholine (ACh). (b) Maximal MCA nitric oxide (NO)‐dependent dilatation (max dilatation_ACh_ − max dilatation_ACh+L‐NAME_. (c) MCA dose–response curve for endothelial‐independent dilatation (EID) to sodium nitroprusside (SNP). (d) The vasoconstrictor response to phenylephrine (PE) in control and UCMS mice with (+nitrite, NO_2_^−^) or without chronic NO_2_^−^ treatment. (a) *P < 0.05 UCMS vs. all other groups; (b) *P < 0.05 between groups. Data are means ± SD, *n* = 19–20 mouse MCA/group. Repeated‐measure ANOVA with a group‐by‐time interaction and Tukey's *post hoc* test.

The endothelial‐dependent dilator (EDD) responses of the MCA in response to increasing concentrations of ACh are summarized in Figure 1. An impaired EDD response to ACh was evident early on (*P *< 0.05, ACh 10^−9^ M) and progressively got worse, resulting in a 60% lower (*P *< 0.001) maximum EDD (ACh 10^−4^ M) in UCMS compared to control mice (Figure 1a). The impaired EDD response was a result of a smaller NO dilatory influence, as indicated by a smaller reduction in EDD in the presence versus absence of the NO inhibitor L‐NAME in the UCMS versus Control group (4.2 µm vs. 15.1 µm) (*P *< 0.001; Figure 1b). The endothelial‐independent dilatation (EID) to SNP and the constrictor response to phenylephrine (PE) did not differ among groups (Figure 1c,d). Chronic NO_2_^−^ treatment prevented the UCMS impairment in EDD to ACh by maintaining NO‐mediated dilatation (Figure 1a,b), as no differences in NO‐mediated EDD among the Con, Con + NO_2_^−^, and UCMS + NO_2_^−^‐treated mice were noted (Figure 1b). Further, chronic NO_2_^−^ treatment did not affect the EID or the PE constrictor responses of the MCA (Figure 1c,d).

**FIGURE 2:**
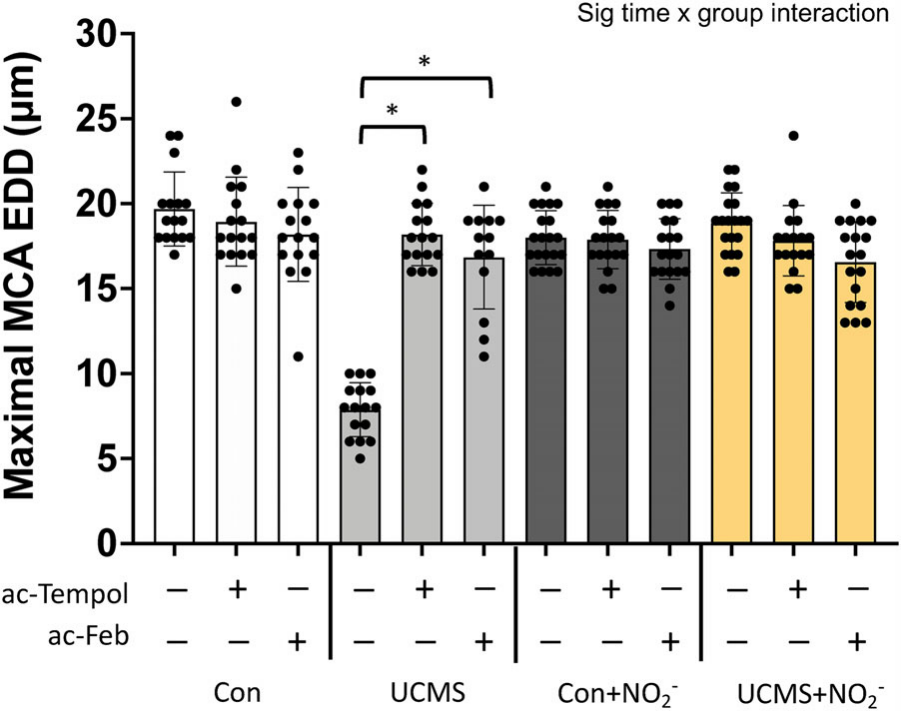
Maximal endothelial‐dependent response and the role of oxidative stress. Maximal endothelial‐dependent dilatation (EDD) to acetylcholine (ACh; 10^−4^ M) with (+) and without (−) acute (30 min) incubation with ac‐Tempol and ac‐febuxostat. *P < 0.05 vs. no incubation experiment within the same group; data are means ± SD, *n* = 16–20 mouse MCA/group. Repeated measured ANOVA with a group‐by‐incubation interaction was performed and when a significant interaction was established, Tukey's *post hoc* test was performed.

To explore the role of oxidant‐mediated impairment of MCA function, we acutely incubated the MCA with Tempol (a superoxide dismutase mimic) or febuxostat (a XO inhibitor) (Figure 2). Both reducers of oxidant levels were equally effective in restoring the maximal EDD of the MCA in the UCMS group. Acute tempol and febuxostat incubation had no additional effect on MCA EDD in the Con, Con + NO_2_^−^, or UCMS + NO_2_^−^ groups.

#### 3.2.1 MCA wall diameters and stiffness

We next examined the extent to which UCMS affected the structural phenotype of the MCA. MCA internal wall diameter did not differ among Con (94 ± 10 mm), UCMS (90 ± 9 mm), Con + NO_2_^−^ (90 ± 12 mm) and UCMS + NO_2_^−^ (91 ± 13 mm) groups. Similarly, wall thickness was not significantly different among Con (14 ± 3 mm), UCMS (14 ± 3 mm), Con + NO_2_^−^ (15 ± 3 mm) and UCMS + NO_2_^−^ (13 ± 2 mm) groups. Further, no significant differences were noted in percentage myogenic tone among groups (Con 34 ± 8%; UCMS 28 ± 12%; Con + NO_2_^−^ 35 ± 8%; UCMS + NO_2_^−^ 32 ± 11%).

### 3.3 Oxidative stress

**FIGURE 3:**
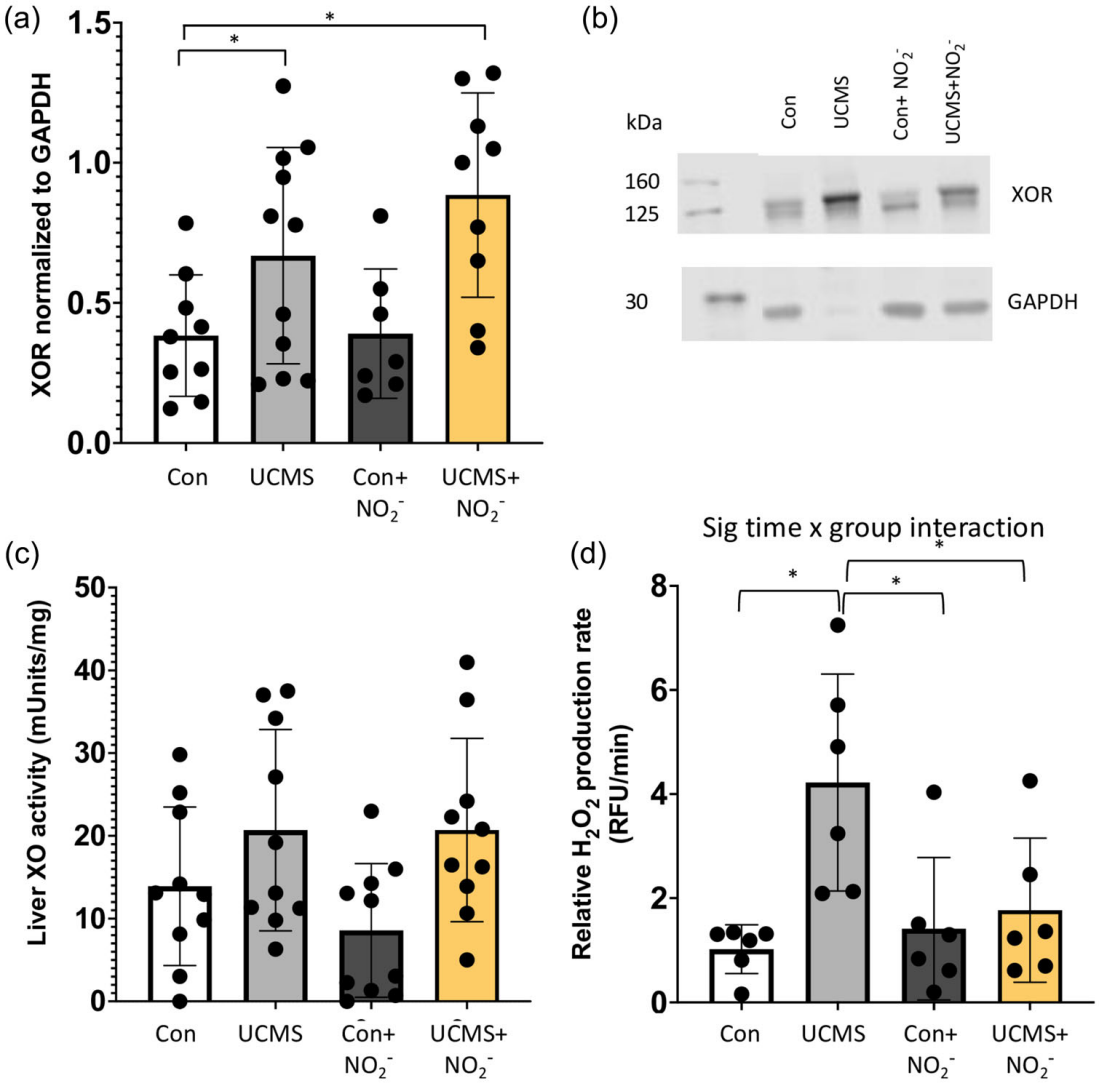
Oxidative stress in the liver. (a) Xanthine oxidase reductase (XOR) concentration was assessed within the liver using western blotting techniques. (b) Representative XOR western blot image. (c) Liver XO activity was measured with HPLC. (d) Liver hydrogen peroxide production was measured via the CBA assay. *P < 0.05; data are means ± SD, *n* = 6–11 mouse samples/group. Two‐way ANOVA with a group‐by‐drug interaction was performed and when a significant interaction was established (d), Tukey's *post hoc* test was performed, but otherwise the main variable effect was reported.

**FIGURE 4:**
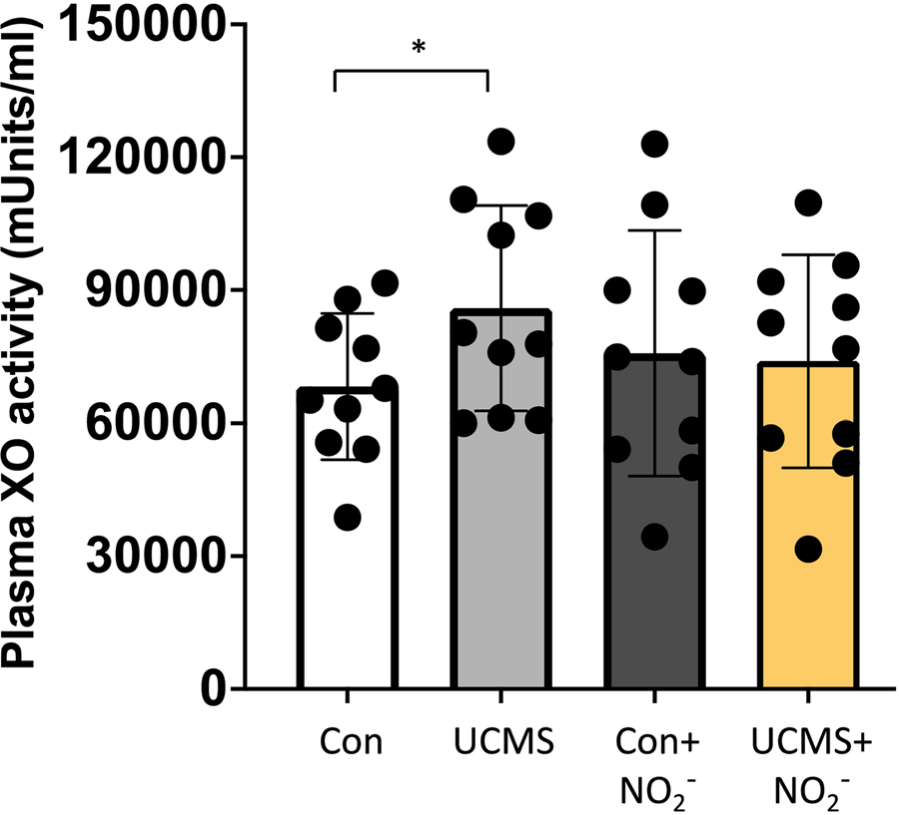
Plasma XO activity. Plasma XO activity was measured by HPLC. *P < 0.05 between groups; data are means ± SD, *n* = 10 samples/group. Two‐way ANOVA with a group‐by‐drug interaction was performed; however, no significant interaction was noted, and thus the main variable effect was reported.

We explored XOR protein abundance in the liver, which was increased (*P *< 0.05) in the UCMS and UCMS + NO_2_^−^ groups compared to control mice (Figure 3a,b). Next, we examined liver XO activity; however, no differences were noted between groups (Figure 3c). As H_2_O_2_ is one of the main end products of the XOR pathway, we used the CBA assay to measure liver H_2_O_2_ production. Liver H_2_O_2_ levels were significantly increased (*P *< 0.05) in the UCMS versus control, Con + NO_2_^−^ and UCMS + NO_2_^−^ groups (Figure 3d). Next, we explored XO activity in the plasma and found a significant increase in the UCMS group (*P *< 0.05) compared to control mice (Figure 4); however, no differences were noted between UCMS + NO_2_^−^ and the Con and Con + NO_2_^−^ groups.

**FIGURE 5:**
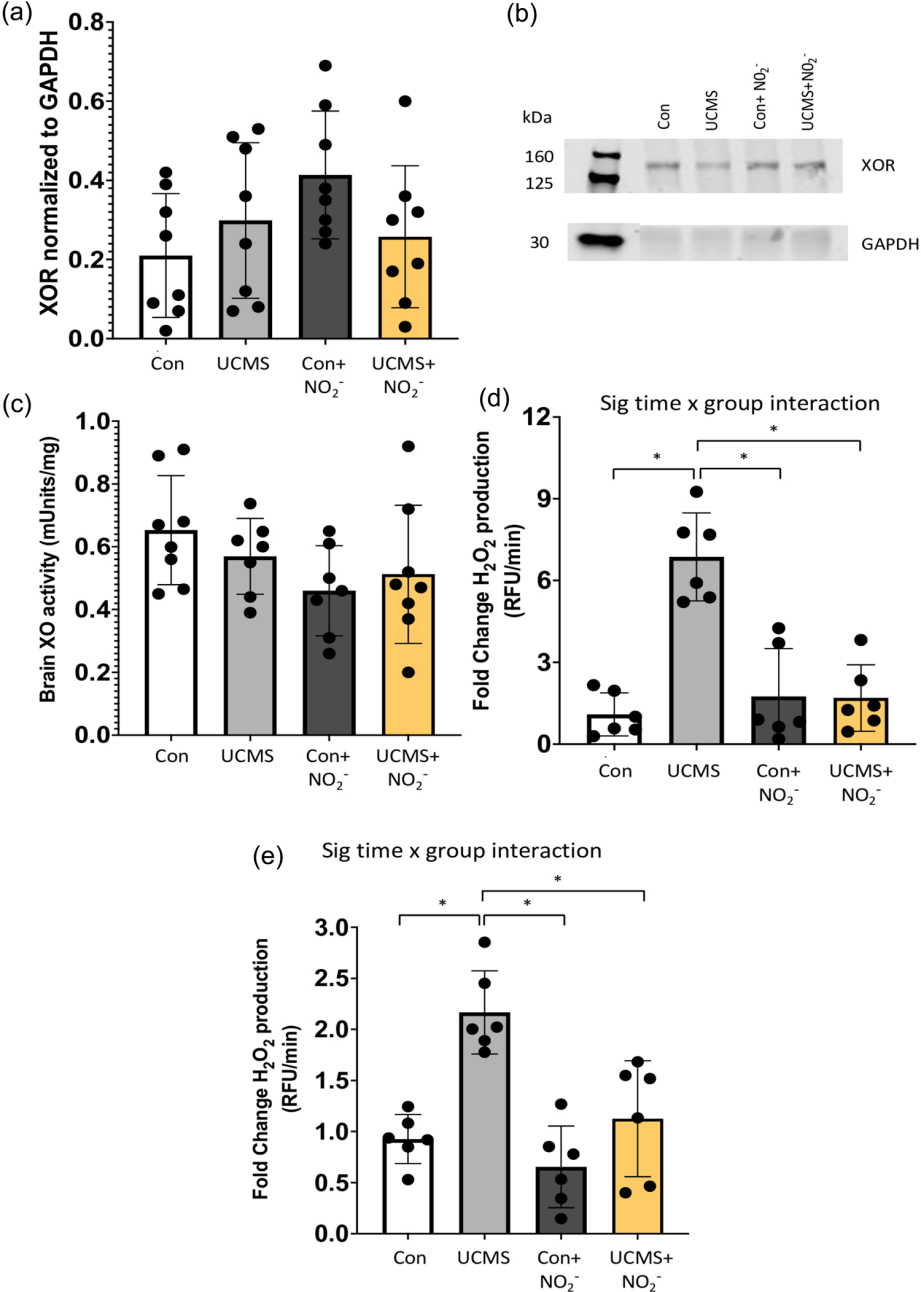
Oxidative stress in the brain and cerebrovasculature. (a) Xanthine oxidase reductase (XOR) level was assessed within the brain using western blotting techniques. (b) Representative XOR western blot image. (c) Brain XO activity was measured with HPLC. (d, e) The CBA assay was used to assess hydrogen peroxide production within whole brain homogenate (d) and isolated brain microvessels (e). Data are means ± SD, *n* = 6–8 mouse samples/group. *P < 0.05 between groups. Two‐way ANOVA with a group‐by‐drug interaction was performed and when a significant interaction was established (d, e) Tukey's *post hoc* test was performed, but otherwise the main variable effect was reported.

Next, we explored XOR in the brain. In brain homogenate, XOR protein levels (Figure 5a) and XO activity (Figure 5b) were similar between groups. However, we did note a 5‐fold increase in H_2_O_2_ production in the UCMS group compared to controls (*P *< 0.05; Figure 5d), which was significantly reduced in the UCMS + NO_2_^−^ group. Next, we explored H_2_O_2_ production in isolated brain microvessels and found a 2‐fold increase in the UCMS group compared to controls (*P *< 0.05; Figure 5e), while treatment with NO_2_^−^ prevented this increase in H_2_O_2_ production (*P *< 0.05).

### 3.4 Behaviour

**FIGURE 6:**
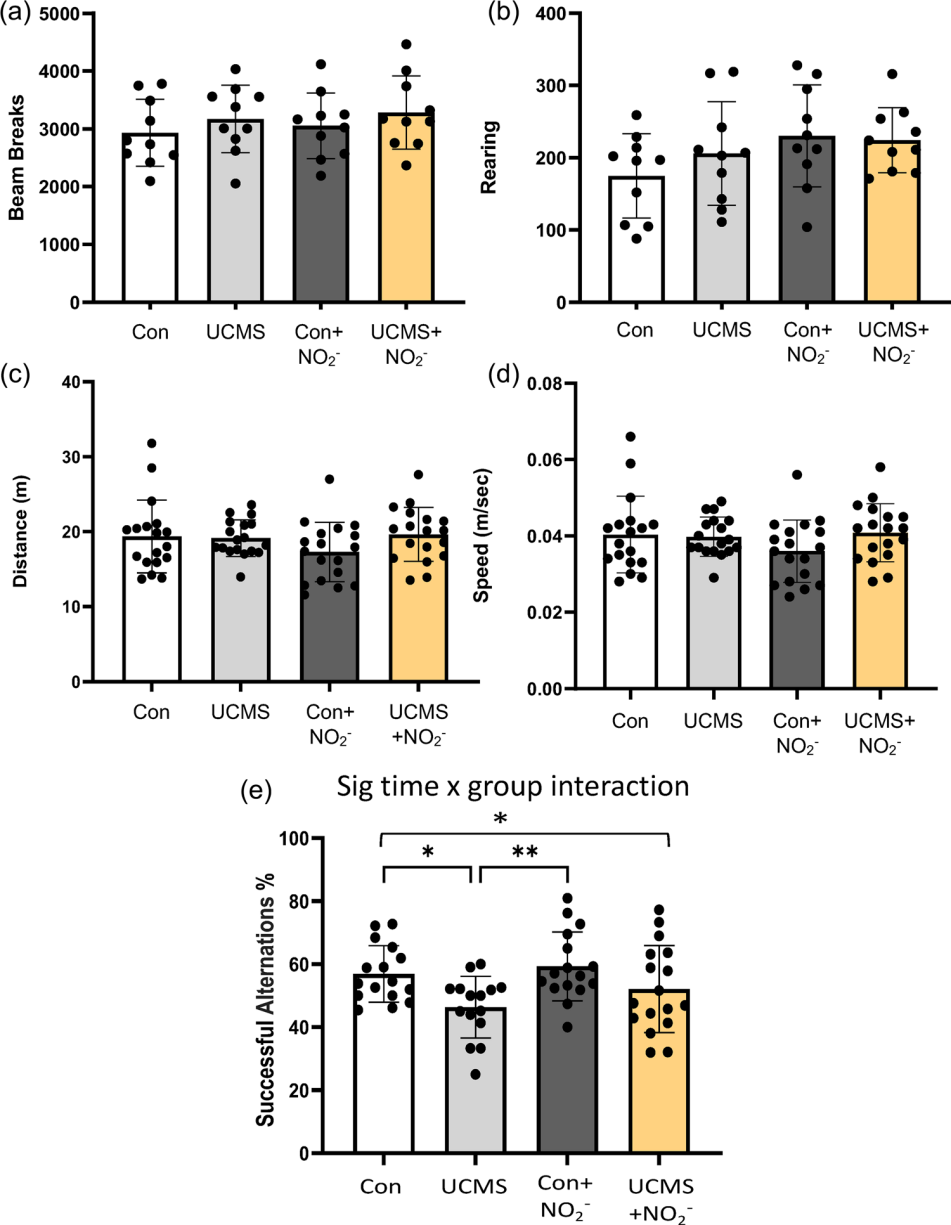
Locomotor and cognitive behaviour with chronic stress. (a, b) An open field test was used to measure locomotor behaviour with total horizontal movements via beam breaks (fine and ambulatory combined) in the arena (a), and the number of vertical beam breaks (rearing; b). (c–e) Spontaneous alternation Y‐maze test was used to measure locomotor behaviour (distance moved (c) and speed (d)) and working memory (e). *P ≤ 0.05 and ** *P* < 0.01 between groups; data are means ± SD, *n* = 10–18/group. Two‐way ANOVA with a group‐by‐drug interaction was performed and when a significant interaction was established (e), Tukey's *post hoc* test was performed.

No differences were noted in the total number of beam breaks (Figure 6a) or the number of vertical beam breaks (rearing) (Figure 6b) between groups in the open field test. We also explored working memory using the spontaneous alternation Y‐maze test and noted a significant reduction in working memory in the UCMS and UCMS + NO_2_^−^ groups compared to controls (Figure 6e). As noted with the open field test and now with the Y‐maze test, the total distance travelled, and the average speed were similar between groups (Figure 6c,d).

## 4 DISCUSSION

The current study aimed to determine if chronic NO_2_ supplementation could prevent the cerebrovascular dysfunction and cognitive decline in mice after 8 weeks of UCMS (Burrage, Coblentz, Prabhu, et al., 2023). We hypothesized that chronic supplementation of NO_2_^−^ would combat the deleterious effects of chronic stress. Several novel observations were found: (1) NO_2_^−^ supplementation during chronic stress prevented the stress‐induced MCA EDD dysfunction by maintaining NO‐dependent dilatation; (2) NO_2_^−^ supplementation prevented the increase in oxidative stress in the liver, brain and cerebrovasculature; and (3) the memory deficits noted with chronic stress were not ameliorated with NO_2_^−^ supplementation.

The chronic stress model has been well‐established in mice and rats to induce significant cerebrovascular dysfunction due to impairments in NO bioavailability mediated partly by a pro‐oxidative microenvironment (Brooks, Branyan, DeVallance, et al., 2018; Burrage, Prabhu, Childers, et al., 2023; Stanley et al., 2014), along with neuropsychiatric alterations, including the development of anxious and depressive‐like behaviour (McEwen et al., 2012; Pego et al., 2008) and cognitive deficits (Cerqueira et al., 2007; Conrad, 2010; Dias‐Ferreira et al., 2009; Dominguez et al., 2019; Jeong et al., 2006; Schwabe et al., 2008). Importantly, these deficits were evident after 8 weeks of chronic stress exposure (Burrage, Prabhu, Childers, et al., 2023; Monteiro et al., 2015). To mitigate the negative effects of chronic stress, we supplemented the water of mice undergoing chronic stress with 50 mg/L of NO_2_^−^. This dose has previously been shown to increase NO levels (Fleenor et al., 2012; Rossman et al., 2021; Sindler et al., 2011). Indeed, our study confirmed that NO_2_^−^ supplementation increased plasma nitrate/nitrite levels in the UCMS group, indicative of enhanced NO levels. In the current study, we observed that NO_2_^−^ supplementation in chronically stressed mice preserved MCA EDD responses, primarily driven by rescued NO‐dependent dilatation, compared to control mice not undergoing UCMS. These data are supported by a human study that showed 7 days of dietary nitrate supplementation greatly increased cerebrovascular reactivity to carbon dioxide in young healthy individuals, which was accompanied by a ∼420% increase in plasma nitrate concentration (Fan et al., 2019). Other human and pre‐clinical studies have shown that NO_2_^−^ supplementation completely reverses age‐related endothelial dysfunction by increasing NO bioavailability (DeVan et al., 2016; Rossman et al., 2021; Sindler et al., 2011). The current study suggests that NO_2_^−^ supplementation improved cerebrovascular function by increasing the circulating levels of NO_2_^−^, which can be rapidly absorbed from the circulation by peripheral tissues where it can be stored in cells until it is needed for use (Sindler et al., 2014). Further, NO_2_^−^ supplementation may have prevented the formation of an oxidative environment; thus, with fewer oxidants generated that can scavenge NO, the bioavailability of NO should increase (Guzik et al., 2002; Newaz et al., 2006; Santhanam et al., 2015) and the MCA produce sufficient dilatation in response to ACh. It is important to note that no differences were noted in smooth muscle cell function either in response of the MCA to SNP or the myogenic tone response. This is consistent with the work of others (Rossman et al., 2021; Sindler et al., 2011) who showed no effect of NO_2_^−^ supplementation on EID in the carotid artery of young or old mice. However, in a mouse model of hypertension, increased matrix metalloproteinases are seen within the smooth muscle cell, and supplementation with NO_2_^−^ prevents this increase in matrix metalloproteinases (Guimaraes et al., 2021). This suggests that NO_2_^−^ supplementation may positively impact smooth muscle cell function if the original stimulus results in smooth muscle cell dysfunction.

Given our previous work showing that XOR may partly mediate cerebrovascular dysfunction and cognitive decline with chronic stress (Burrage, Prabhu, Childers, et al., 2023), we explored XOR as a potential mechanism of action of NO_2_^−^ supplementation. While XOR produces oxidants under pro‐inflammatory conditions, impairing endothelial cell function (Cantu‐Medellin & Kelley, 2013; Kelley et al., 2010), providing an alternative substrate (i.e., NO_2_^−^) drives XOR reduction of NO_2_^−^ to NO (Cantu‐Medellin & Kelley, 2013; Kelley, 2015; Li et al., 2005, 2009) and thus contributes to vasodilatation and vascular homeostasis. As the liver is the main site of XOR production, activity and circulating XO (the oxidized form of XOR) (Harmon et al., 2019; Parks & Granger, 1986), we assessed the impact of NO_2_^−^ supplementation on the oxidative environment of the liver. We found that production of H_2_O_2_ (one of the typical by‐products of XOR) in the liver was increased in the UCMS mice, but the mice receiving NO_2_^−^ supplementation had H_2_O_2_ levels similar to control mice. Concurrently, we found that UCMS mice, regardless of supplementation, had increased liver XOR protein levels. These data suggest that the mice undergoing chronic stress alongside NO_2_^−^ supplementation display altered product formation that prevents the induction of a pro‐oxidative environment within the liver. The liver XOR has been implicated in facilitating XO to the circulation, where it can reach sites distal to the liver (like the cerebrovasculature), resulting in arterial dysfunction. Mice exposed to chronic stress displayed an increase in plasma XO activity; however, supplementation with NO_2_ prevented this increase in plasma activity. Interestingly, others have noted that NO produced via XOR has the innate ability to limit XOR activity, which may explain the lack of plasma XO activity in the NO_2_^−^ supplemented group that underwent chronic stress (Damacena‐Angelis et al., 2017; Ichimori et al., 1999). Given the proximity of the brain tissue with the cerebrovasculature, we explored XOR protein and XO activity in brain homogenate. No differences were noted in XOR protein or XO activity in the brain. However, H_2_O_2_ was significantly increased in the brain tissue of UCMS mice and significantly reduced in the UCMS + NO_2_^−^ mice. Importantly, this persisted within the cerebral vessels, with UCMS mice having a 2‐fold higher production of H_2_O_2_; however, NO_2_^−^ supplementation prevented this increase in H_2_O_2_. Taken together, these data suggest that NO_2_^−^ supplementation may have limited the oxidant production in the liver, brain and cerebral circulation, allowing for abundant NO production sufficient to maintain EDD of the MCA. This is supported by recent reports (Kelley, 2015; Ortiz de Zevallos et al., 2022) and various studies (Burrage, Prabhu, Childers, et al., 2023; Tian et al., 2020) showing that XOR can act as a NO_2_^−^ reductase with implications for vascular NO and ROS homeostasis, particularly in conditions associated with eNOS dysfunction. Peleli et al. (2016) showed that in eNOS knockout mice, XOR activity was upregulated compared with wild‐type and that an acute dose of nitrate resulted in greater plasma NO_2_^−^ levels in eNOS^−/−^ compared with wild‐type mice while concomitantly reduced O_2_^●−^, which was abolished by the specific XOR inhibitor febuxostat. Further, the eNOS^−/−^ mouse livers showed higher NO_2_^−^ reducing capacity than those of the wild‐type mice, which was again attenuated by febuxostat.

Other potential pathways by which NO may reduce oxidative stress include the ability for NO_2_^−^ supplementation to reduce whole‐cell and mitochondrial ROS bioactivity, thus improving endothelial function (Rossman et al., 2021). Others have shown that increased NO levels can improve blood flow, indirectly reducing oxidative stress by delivering more oxygen to tissues while inhibiting inflammatory processes (Wightman et al., 2015), thereby leading to an improved environment for endothelial function. It is also important to note that nitrate supplementation can reduce NADPH oxidase activity in animal models of disease (Gao et al., 2015) and increase superoxide dismutase enzyme activity (Sindler et al., 2011), which may have also contributed to a reduction in oxidative stress. Along with the mitochondrial production of oxidants, both XOR and NADPH oxidase are the major sources of free radical formation. Furthermore, if a normal oxidative environment is preserved, and thus eNOS is not uncoupled, then eNOS can also contribute to NO generation by reducing nitrite to NO, which is known to occur under certain conditions like hypoxia (Webb et al., 2008). As such, these additional pathways may be responsible, in part, for the positive effects of chronic NO_2_^−^ supplementation on cerebrovascular function. Future studies should address the role of XOR as the potential main factor contributing to enhanced NO bioavailability and limited oxidative stress production in the context of chronic stress by using XOR inhibitors or XOR‐deficient mouse models.

Our recent work has identified the potential clinical consequences of chronic stress exposure on cognitive function, specifically working memory decline in mice exposed to chronic stress (Burrage, Coblentz, Prabhu, et al., 2023). Interestingly, NO_2_^−^ supplementation did not completely prevent this decline in the working memory of chronically stressed mice. In human studies, the impact of NO_2_^−^ supplementation on cognitive function has received mixed results. In some studies, NO_2_^−^ supplementation positively affects cognitive function (Justice et al., 2015; Wightman et al., 2015). However, in other studies, NO_2_^−^ supplementation has no impact (Clifford et al., 2019; Kelly et al., 2013). Further, a recent systematic review and meta‐analysis of randomized clinical trials examining the effect of inorganic nitrate or nitrite supplementation on cognitive function showed that nitrate and NO_2_^−^ supplementation did not modify cognitive function (Clifford et al., 2019). These mixed results may be due to differences in either the dose of NO_2_^−^ supplementation or the specific cognitive domain that was assessed. However, other forms of memory may have been impacted that we did not assess. Indeed, a limitation of the study is that we only focused on the Y‐maze spontaneous alternation test, widely used in various animal models to assess short‐term working memory. Indeed, Alqurashi et al. (2022) showed that although working memory was the first memory to be impaired after 5–6 weeks of UCMS, later states of UCMS affected all types of memory (recognition, long‐term spatial, and reference). Future work should explore a battery of exploration, social and cognitive tests.

### 4.1 Conclusions

The present study investigated whether NO_2_^−^ supplementation, when administered concurrently with exposure to chronic stress, could protect cerebrovascular function and cognitive health. We showed that NO_2_^−^ prevented the development of a pro‐oxidative environment within the liver, brain and cerebrovascularture, and prevented the cerebrovascular dysfunction associated with chronic stress. However, NO_2_^−^ supplementation did not rescue the stress‐induced deficits in working memory. Given the beneficial effects of NO_2_^–^ supplementation on improving vascular EDD in older mice and humans (Fleenor et al., 2012; Sindler et al., 2014, 2011), it would be valuable to explore whether NO_2_^−^ supplementation would have similar protective effects in aged mice exposed to UCMS as observed in the younger mice in this study.

## AUTHOR CONTRIBUTIONS

The study was performed in PDC and EEK laboratories. Paul D Chantler, Emily Burrage, Saina S. Prabhu, Nicole M. Eminhizer, Ryan Childers, Evan R. DeVallance, Randall W. Bryner and Eric E Kelley: conceptualized and designed the study. Paul D Chantler, Emily Burrage and Eric E Kelley: undertook experiments and analysed data. All authors have read and approved the final version of this manuscript and agree to be accountable for all aspects of the work in ensuring that questions related to the accuracy or integrity of any part of the work are appropriately investigated and resolved. All persons designated as authors qualify for authorship, and all those who qualify for authorship are listed.

## CONFLICT OF INTEREST

None declared.

## Data Availability

All the data that support the findings of this study are presented in Table 1 and Figures 1, 2, 3, 4, 5, 6.
